# Event-Specific Risk Factors Predicting Episodes of Unprotected Anal Intercourse with Male Nonregular Partners among Men Who Have Sex with Men Using Case-Crossover Study Design

**DOI:** 10.1155/2014/475195

**Published:** 2014-07-20

**Authors:** Jinghua Li, Joseph T. F. Lau, Jing Gu, Chun Hao, Coco H. Y. Lai

**Affiliations:** ^1^School of Public Health and Primary Care, Faculty of Medicine, The Chinese University of Hong Kong, Shatin, Hong Kong; ^2^CUHK Shenzhen Research Institute, Shenzhen 518057, China; ^3^Centre for Medical Anthropology and Behavioral Health, Sun Yat-sen University, Guangzhou 510080, China; ^4^Centre for Health Behaviors Research, School of Public Health and Primary Care, Faculty of Medicine, The Chinese University of Hong Kong, 5/F., School of Public Health, Prince of Wales Hospital, Shatin, New Territories, Hong Kong; ^5^School of Public Health, Sun Yat-sen University, Guangzhou 510080, China

## Abstract

This study investigated event-specific factors that determine episodes of unprotected and protected anal intercourse (UAI and PAI) among 215 men who have sex with men (MSM), who used condoms inconsistently with nonregular partners (NRP) in the last six months, in Hong Kong. A case-crossover study design was used. Lower likelihood of episodes involving UAI with NRP was associated with (1) five partner attributes (NRP were <35 years old, at least three previous anal sex experiences with the NRP, perception that participant and the NRP had asymmetrical sexual experience, perception that the NRP was feminine, and liking toward the NRP; OR = 0.16–0.52), (2) six situational variables (the participant having had UAI with another man in the last week, having discussed condom use, perception that the NRP liked to use condom, partner's suggestion to have PAI, participant's suggestion to have PAI, and participant's plan to use condoms; OR = 0.11–0.39), and (3) four environmental/setting variables (condoms already placed at the venue, display of condom use promotion materials, participant's possession of a condom, and the NRP possessed a condom; OR = 0.27–0.45). HIV prevention targeting MSM should focus on event-specific protective factors, which may be different from those obtained from studies distinguishing condom users versus nonusers.

## 1. Introduction

In many Asian cities, HIV prevalence among men who have sex with men (MSM) has been increasing sharply [1]. In China, a 61-city study documented HIV prevalence of 4.9% in 2009 [2]. The incidence of HIV was high in China, 5.1 and 5.4 per 100 person-years in Nanjing [3] and Shenyang [4], respectively. In 2008, the HIV prevalence among MSM in Hong Kong was 4.3% [5]. Specifically, 84.6% of the MSM in Hong Kong had had sex with nonregular partners (NRP) in the last six months; of those MSM, 27.9% had had unprotected anal intercourse (UAI) with a NRP [6].

Globally, high percentages of MSM have NRP, and UAI is often involved [7–9]. Factors associated with UAI are often reported and serve as basis for forming HIV interventions to reduce UAI [10, 11]. The factors that specifically apply to NRP among MSM include low education level [12], low level of HIV-related knowledge [12], recruitment of male sex partners at gay venues [9], lack of exposure to HIV prevention services [7], and concern about acquiring sexually transmitted diseases [8]. Factors associated with UAI with NRP among MSM in Hong Kong include perceived chance of contracting HIV, having had anal sex with more than six partners, perceived nonavailability of condoms, and sourcing NRP from brothels [6]. In these studies, the participants who were MSM were divided into a group of men ever had had UAI and never had had UAI in the past month or past few months. Cross-sectional analysis is commonly used to distinguish between these two groups.

Another research question is, however, important to HIV prevention. Instead of what mentioned above, the question focuses only on the group of inconsistent condom users, that is, those who sometimes had UAI and sometimes had protected anal intercourse (PAI). The research question is “Why do these inconsistent condom users sometimes use condoms and sometimes not?” or “Are the situations different when UAI occurs and does not occur?” This new research question cannot be answered by traditional cross-sectional analysis and logistic regression analysis, as it involves only one group of MSM (those who were inconsistent condom users). It can, however, be answered by a relatively new study design, the case-crossover design, which can be used to identify situational predictors of UAI within the same individuals, who sometimes but not always use condoms during anal intercourse (inconsistent condom users). In our context, the design compared presence of situational factors in two episodes of anal intercourse, the last episode of UAI and the last episode of PAI. The matched (conditional) logistic regression method is a standard method to analyze such data. In our case, a pair of episodes of UAI and PAI of the same individual is used as unit of analysis [13, 14]. Hence, it allows researchers to investigate various event-specific risk factors to distinguish episodes of UAI from episodes of PAI.

The literature shows that the case-crossover study design is able to delineate the temporal relationship between variables of interest and has better control for confounding factors (e.g., participants' characteristics), since the information is obtained from the same individuals [15]. Thus, it is a compelling approach for investigating a causal pathway [16] and can minimize biases due to measured and unmeasured time-invariant potential confounders since every sampled individual serves as his own control [17]. The case-crossover analysis has important implications in HIV prevention, as it draws health workers' attention to situational factors that lead to UAI among inconsistent condom users, a group that may benefit more than consistent condom users from HIV prevention programs. Although the case-crossover design is a relatively new method, it is well established and has been used in studying different types of health-related behaviors (e.g., alcohol and drug use, first-time sexual encounter, and sex at home). A dearth of studies has, however, used this type of design to answer the aforementioned research question about HIV prevention.

The present study applied the case-crossover study design to investigate whether three broad types of event-specific factors are predictive of episodes of UAI among MSM who were inconsistent condom users during anal sex with male NRP in Hong Kong: (1) factors related to the sex partner's attributes (e.g., age and physical appearance), (2) situational factors (e.g., appraisal of the risk of the NRP, communication with the NRP about condom use, sexual compulsivity, and use of alcohol), and (3) environmental/settings factors (e.g., timing and location of the sex episode, availability, and possession of condoms).

## 2. Methods

### 2.1. Study Design

Inclusion criteria were (1) Hong Kong Chinese men from 18 to 60 years old (those holding a Hong Kong identification card), who have (2) had both at least one episode of UAI with a NRP and at least one episode of PAI with a NRP (i.e., an inconsistent condom user) in the last six months. A mapping exercise was conducted by the government and identified 12 gay bars and 16 gay saunas in Hong Kong. Approval was obtained from six gay bars and ten gay saunas.

The venue-based interviews were administered by a group of experienced and trained peer interviewers. Participants were also recruited from two public beaches that were favored by gay men. This combined method has been used to recruit MSM study participants [18–20]. Peer interviewers visited these venues at different time slots during weekdays and weekends. They briefed prospective participants about the study and invited them to join the study. Venue-based sampling was performed from September 2010 to June 2011. Several screening questions were asked to establish the participants' eligibility and verbal informed consent was obtained before the face-to-face interview commenced in settings with privacy ensured. Interviewers signed a form pledging that they had explained information contained in the information sheet clearly to the participant. Upon completion of the interview, participants were given a coupon of HK$50 (about 6 USD) cash value, to compensate their time spent for the interview.

In addition to venue-based sampling, some participants were recruited through the internet, since some MSM did not attend gay venues but are accessible through the internet. Previous studies have shown that many MSM recruit friends and sexual partners from the internet make recruiting this population an important step for understanding HIV-related risk behaviors. The mixed method has been used in many published studies on sexual health of MSM, including a number of local studies [18, 20] and others conducted outside Hong Kong [21, 22], and enabled us to recruit MSM of different characteristics. Banners were posted on four gay websites, which were frequently visited by MSM in Hong Kong. The websites have functions of chat rooms, partner finding, and provision of information (addresses of local gay venues) and are well known to MSM in Hong Kong. Since the recruitment method was different from that of the venue-based sampling, verbal informed consent was not feasible for the internet-based recruitment which was not administered by fieldworkers. Instead, participants were provided with information at the beginning of the online questionnaire, which briefed them about the voluntary nature, background, and purposes of the study and told that the return of the completed internet-based questionnaire to the researchers implied provision of their informed consent [18–20]. The internet-based participants then self-administered the anonymous internet-based questionnaire, which was identical to the one used in the venue-based survey. Participants were able to claim a supermarket coupon of HK$50 cash value by providing a mailing address (without including their names). The envelops did not include any name nor information about HIV; the mailing of coupons as an incentive for completing surveys has been performed in other published studies on MSM [23, 24]. We cross-checked that there were no overlapping addresses provided to us. A total sample size of 215 was achieved (151 venue-based interviews and 64 internet-based questionnaires).

### 2.2. Measures

#### 2.2.1. Background Characteristics of the Participants

Data on sociodemographic variables, sexual orientation, and the mode of recruitment were collected.

#### 2.2.2. Last Episodes of UAI and PAI with Male NRP

Participants reported the presence or absence of an event-specific factor during the most recent episode of UAI with a NRP and the most recent episode of PAI with a NRP. (e.g., “*Did you use alcohol before the last episode of UAI with a male NRP*?” or “*Did you use alcohol before the last episode of PAI with a male NRP*?”). As mentioned, these data were analyzed in paired data of the same individuals. Operationally, a NRP was defined as “one whom you met casually for the purpose of having sex but did not involve money.”

#### 2.2.3. Event-Specific Factors Related to Attributes of the Nonregular Male Sex Partners (NRP)

Information collected on NRP included (1) sociodemographic characteristics, (2) sexual experiences, (3) style of the partner (i.e., physical appearance being masculine, feminine or neutral, and perceived personality of the NRP being assertive, passive, or in-between), and (4) degree of liking (i.e., to which extend is the NRP attractive to the participant, how much the participant liked the NRP, and how much the participant perceived that the NRP liked him, on a scale from 1 “did not like” to 10 “liked very much”) and relationship with the NRP (i.e., how the participant rates the relationship with the NRP using a 5-point Likert scale from “very good” to “very bad”).

#### 2.2.4. Situational Variables

Information gathered about the situation surrounding UAI and PAI included (1) the participant's spirit prior to the episode of anal sex (mood (how did the participant feel prior to having anal sex?), tiredness (whether the participants felt tired), and whether the participants felt nervous), (2) risk assessments (e.g., perceived risks of HIV and STI of the NRP), (3) communication and planning about condom use (e.g., discussion with the NRP about condom use and condom use being suggested by the participant), (4) sexual behavioral variables (e.g., sexual compulsivity and sexual position in the last episode of UAI and PAI and sexual dysfunctions of no sexual desire, erectile dysfunction, premature ejaculation, and pain during intercourse during the previous month), and (5) substance use before anal intercourse took place (alcohol, potency drugs, and psychoactive substances).

#### 2.2.5. Event-Specific Environmental and Settings Variables

These variables included (1) timing and location of the sexual episodes (e.g., Hong Kong or overseas, weekend or weekday, at home or not at home), (2) perceived availability of condoms and display of reminders promoting condom use, and (3) physical conditions (i.e., whether the anal sex took place in a dark environment, whether there was shower facilities, and whether the hygiene of the place was relatively poor).

### 2.3. Statistical Analysis

As mentioned, data of the case-crossover design were organized and analyzed in pairs (i.e., presence or absence of the situational factors in the last UAI and last PAI episodes) which were matched within the same individuals. By matching, it means pairing of data. The presence or absence of a situational factor referring to the UAI episode was matched to (or paired with) those of the PAI episode. The data set was rearranged so that there were 215 pairs of data. For instance, for the situational factor of alcohol use, the paired data for the UAI and PAI episodes could be (yes, yes), (yes, no), (no, yes), and (no, no). It therefore refers to internal matching of all situational factors within the same individual. Data of all situational factors were organized and analyzed. To account for such pairing, conditional logistic regression, a standard method for the case-crossover design [13, 14], was used to assess the significance of the associations between the studied event-specific risk factors and occurrence of UAI. Matched odds ratios (OR) and their 95% confidence intervals (CI) were presented. Since potential confounders of individuals' characteristics have been removed by pairing data of situation factors within the same individuals, such analyses do not need to adjust for participants' background characteristics [13]. Univariate matched OR are hence reported for each variable. The software PROC PHREG (SAS Institute, Cary, NC, release 9.2) was used. All other statistical analyses were performed using SPSS 18.0 (SPSS Inc., Chicago, IL). All statistical tests involved were two-tailed and a *P* value <0.05 was considered statistically significant.

## 3. Results

### 3.1. Descriptive Statistics

About 3/4 (74%) of the participants were 35 years old or younger; 62.8% had attended a college or university; 87.4% identified as homosexual (see Table 1). No significant differences in sociodemographic variables were found between participants recruited from gay venues and via the internet (*P* > 0.1, see Table 1).

The majority of NRP were of 35 years old or younger (about 70%), Hong Kong residents with Hong Kong identification cards (>80%), and perceived by most participants (about 87%) as attractive. About 50% of the NRP were described by the participant as having appearance which looked masculine and perceived to have an assertive personality. Over half (57.7%) of the NRP met the participant on the same day and 68.6% had never had anal sex with the participant in the past (see Table 2).

Since there are many situational and environmental factors included in this study, we only describe the frequency distributions of those variables that were found to be significant in the conditional logistic regression that are described in Table 5, while the frequency distributions of all factors are listed in Tables 3 and 4. The prevalence of presence of the significant situational and environmental factors in the UAI episode and PAI episode was being in an energetic or very energetic state prior to the episode of anal sex (UAI episode: 42.4%; PAI episode: 46.0%), nervous feeling prior to the episode of anal sex (UAI episode: 57.2%; PAI episode: 50.3%), having had UAI with another man one week prior to having sex with the NRP of the last episode anal sex (UAI episode: 14.4%; PAI episode: 20.9%), perception that the NRP would be unlikely to (or never) use condoms if they were going to have anal sex with other men (UAI episode: 49.8%; PAI episode: 41.4%), discussion about condom use prior to having had anal sex with the NRP (UAI episode: 21.4%; PAI episode: 37.2%), perception that the NRP would like to use condom (UAI episode: 15.8%; PAI episode: 50.7%), suggestion made by the NRP to use condom (UAI episode: 16.3%; PAI episode: 34.0%), suggestion made by the NRP to have UAI (UAI episode: 24.2%; PAI episode: 13.0%), plan made by the participant to use condom (UAI episode: 54.9%; PAI episode: 81.9%), participant's suggestion to use condom (UAI episode: 20.0%; PAI episode: 43.7%), participant's suggestion to have UAI (UAI episode: 15.3%; PAI episode: 6.5%), anal sex took place during a weekday (UAI episode: 67.9%; PAI episode: 54.0%), condoms already placed at the venue where that anal intercourse took place (UAI episode: 70.7%; PAI episode: 81.9%), display of HIV prevention materials (e.g., posters) at the venue where anal sex took place (UAI episode: 41.4%; PAI episode: 49.3%), condoms possessed by the participant (UAI episode: 29.3%; PAI episode: 37.2%), and condoms possessed by the NRP (UAI episode: 11.6%; PAI episode: 23.3%).

### 3.2. Factors Distinguishing Episodes of UAI from Episodes of PAI

Higher likelihood of episodes involving UAI with NRP was associated with (1) one attribute of the NRP (perceived assertive or neutral personality (OR = 2.18, 95% CI = 1.07 to 4.45 and OR = 4.30, 95% CI = 1.81 to 10.17; reference group perceived passive personality)), (2) five situational variables (feeling tired before having anal sex (OR = 2.73, 95% CI = 1.07 to 7.00), feeling nervous before having anal sex (OR = 3.32, 95% CI = 1.57 to 7.06), perception that the partner was unlikely to or would never use condoms during anal sex with other men (OR = 2.29, 95% CI = 1.22 to 4.28), having the partner suggest UAI (OR = 4.00, 95% CI = 1.84 to 8.68), and having the participant suggest UAI (OR = 2.90, 95% CI = 1.41 to 5.95)), and (3) one environmental/setting variable (the anal sex episode took place on a weekday (OR = 2.50, 95% CI = 1.49 to 4.20)) (see Table 5).

Significantly lower likelihoods of episodes involving UAI with NRP were associated with (1) five NRP attributes (episodes involving NRP of ≤35 years old or of unknown age (OR = 0.52, 95% CI = 0.28 to 0.97 and OR = 0.29, 95% CI = 0.12 to 0.70; reference group: ≤25 years old), having had anal sex at least three times previously with the NRP (OR = 0.28, 95% CI = 0.10 to 0.75), perception that he and the NRP possessed asymmetrical duration of sexual experience (OR = 0.47, 95% CI = 0.27 to 0.79), perception that the NRP looked feminine (OR = 0.16, 95% CI = 0.04 to 0.57), and higher degree of liking toward the NRP (score ≥ 7 out of 10) (OR = 0.19, 95% CI = 0.04 to 0.81)), (2) six situational variables (perception that the participant had had UAI with another men in the last week (OR = 0.39, 95% CI% = 0.18 to 0.85), discussion about condom use prior to anal sex (OR = 0.29, 95% CI = 0.16 to 0.53), perceived partner's liking in condom use (OR = 0.11, 95% CI = 0.05 to 0.21), the partner's suggestion to have PAI (OR = 0.30, 95% CI = 0.17 to 0.52), the participant's suggestion to have PAI (OR = 0.19, 95% CI = 0.10 to 0.35), and the participant planned to use condoms (OR = 0.16, 95% CI = 0.08 to 0.30)), and (3) four environmental/setting variables (condoms already placed at the venue (OR = 0.40, 95% CI = 0.22 to 0.71), display of condom use promotion materials (OR = 0.40, 95% CI = 0.21 to 0.76), the participant possessed a condom (OR = 0.45, 95% CI = 0.24 to 0.85), and the NRP possessed a condom (OR = 0.27, 95% CI = 0.13 to 0.56)) (Table 5).

None of the variables belonging to the blocks of variables related to particular sexual behaviors, substance use, and the physical environment were found to be statistically significant in the conditional logistic regression analysis.

## 4. Discussion

This study presents in detail some situational factors that were associated with episodes of UAI with NRP. Some characteristics of the sexual episodes are interesting. A minority of the NRP was seen as HIV positive or at high risk of HIV infection but the majority of the NRP was seen to have other sex partners and not using condoms with such partners. Also, about 1/5 and 1/7 of the participants had had UAI with another male partner during the week prior to the last episode of PAI and UAI with the NRP, respectively. Discussion about condom use with the NRP did not happen frequently. Condom availability appeared to be a barrier to engaging in PAI, as participants reported that neither they nor their NRP typically carried condoms with them. Overall, the participants reported being at high risk of HIV infection but not in a good position to prevent HIV infection.

Previous sexual experiences mattered in the prediction of UAI with NRP. The risk of UAI was lower for episodes involving NRP if the participants had had anal sex at least three times with that NRP. This suggested that it might be easier for the participants to negotiate condom use with a NRP that he had had sex with for several times in the past. Episodes involving asymmetrical duration of sexual experiences between the participant and the NRP were also associated with lower risk of UAI. It is possible the participant or the NRP would be more cautious when facing a sex partner who is more or less sexually experienced. The exact reasons behind the UAI are not clear. As a limitation, we did not separate higher versus lower level of sexual experience as compared to that of the NRP.

Those NRP perceived as being feminine, being perceived as having a less assertive personality, or being liked by the participant were less likely to engage in UAI. Thus, HIV prevention education might give special attention to MSM who look masculine or those who have a more assertive personality. There is evidence that, among consistent and inconsistent condom users, being sexually attracted to a partner is a risk factor for unprotected sex [25]; however, the findings of the current study are not consistent with this, suggesting that factors discriminating between episodes of UAI and PAI with NRP among MSM who are inconsistent condom users may be different from those discriminating between consistent and inconsistent condom users among all MSM.

It is important to note that almost all the situational factors related to condom negotiation and the perception that the partner liked to use condoms were significant protective factors of episodes of UAI with NRP, corroborating with results obtained from studies discriminating between consistent and inconsistent condom users among all MSM [26]. Therefore, HIV prevention attempting to reduce UAI among MSM who are inconsistent condom users with NRP should focus on situational factors. As the perception that the NRP disliked using condoms with other male sex partners was also a significant factor, MSM with NRP should be reminded not to assume that the NRP would not want to use condoms, as such perceptions may be inaccurate while preference on condom use is relative and changeable. Instead, they should be encouraged to discuss condom use explicitly and consistently with the NRP.

Of equal importance is the protective effect of condom availability, which corroborates literature discriminating consistent versus inconsistent condom users among MSM [6, 27]. The results of the current study have practical implications, in that it seems useful to place and to distribute condoms at the venue where anal sex commonly takes place. The participants' possession of condoms is another protective factor against episodes of UAI with NRP. HIV prevention programs should therefore attempt to create a norm that it is socially acceptable and likely beneficial for sexually active MSM to always carry a condom with them. Small, stylish, and high quality capsules (e.g., key chains) to carry condoms can be designed and distributed to MSM. Many intervention programs, such as those involving condom distribution exercises, have tried to increase condom availability [28] but very few programs have reminded MSM to always carry condoms with them. This attempt would be a complement to other efforts promoting condom availability among MSM having anal intercourses with NRP.

In addition, based on the current results, seeing reminders promoting condom use displayed at venues where sexual encounters occur might reduce the risk of UAI with NRP. Venues such as gay saunas and hotels should therefore ensure that there is a supply of condoms and display such posters or reminders that remind MSM to use condoms at that venue. These posters may thus serve as cue to action, which is a construct of the Health Belief Model that has shown to be significantly associated with many health-related behaviors, including condom use [29]. In HIV prevention programs, MSM who are inconsistent condom users may also be provided with reminders about every-time condom use, items that they can wear (e.g., pins and bracelets) or be placed at home (e.g., photo frame, towels, and bathroom utilities) that can serve as cues to action to avoid UAI. They can also choose their own items, which may not need to be specifically related to HIV prevention to avoid stigmatization.

One surprising result from the current study is that sexual episodes taking place during a weekday and tiredness and nervous feelings prior to anal sex with NRP were risk factors of UAI with NRP. MSM who are inconsistent condom users with NRP should be reminded about the increased risks of UAI when they are exposed to these risky situations. In practice, more venue-based (e.g., gay saunas) HIV prevention outreach activities could be conducted during weekdays. Special midweek reminders may also be flagged at such venues. MSM should also be reminded of the importance of foreplay to reduce any nervous feelings prior to anal intercourse. At gay saunas, for instance, MSM may be advised to have a massage before engaging in sexual intercourse so as to reduce tiredness and anxiety. These are ideas that have not yet been investigated, as many of these factors leading to UAI have not been investigated in previous studies of consistent and inconsistent condom users.

Previous studies have shown that the use of alcohol and substances was associated with UAI but mixed results were obtained [30, 31]. It is interesting that our data did not find alcohol use to be a risk factor of episodes of UAI with NRP. The prevalence of substance use was rather low and no significant associations with UAI were found. Other potentially important factors were also found to be nonsignificantly related to UAI, including perception that the NRP have contracted HIV/STI and perceived number of sex partners of the NRP. Such findings are informative, as they remind healthcare workers that MSM may have UAI with NRP even if they are aware that such intercourses may involve high risk of HIV/STI transmission. It implies that HIV prevention attempting to raise relevant risk perceptions, a common approach, would be unlikely to be effective in prevention programs targeting MSM who are inconsistent condom users. The associations between risk perception and condom use reported in studies discriminating consistent and inconsistent condom users were mixed [32].

Furthermore, while variables related to the physical environment (e.g., shower facilities and hygiene), sexual compulsivity, sexual positions were found to be significantly associated with UAI in traditional studies discriminating consistent and inconsistent condom users [33–37], such variables were nonsignificant in this study. This discrepancy confirms the need to consider a different set of factors for designing HIV prevention campaigns targeting MSM who are inconsistent condom users with NRP.

In sum, factors that have been shown to discriminate between consistent condom users and inconsistent condom users may not be applicable to understanding why MSM who are inconsistent condom users sometimes but not always use condoms during anal sex with NRP. It is important to ensure that those distinguishing factors not be overemphasized when designing HIV interventions. Instead, situational factors such as those found in this study should be considered. In addition, more research in this area is warranted.

The case-crossover study design has the benefit of minimizing between individual confounders as each individual serves as his own control [16]. The study, however, also has some limitations. First, the data used was based on self-reported ones and was hence subject to recall bias. Second, the time since the last episodes of UAI and PAI may have varied between individuals; the time interval between the UAI and the PAI was not uniform across individuals. Third, there may have been multiple incidences of UAI and PAI within the participants. The association with UAI may vary, depending on which pair of episodes were selected. We chose only the most recent pair for data analysis and assumed high consistency in characteristics associated with different episodes of UAI and different episodes of PAI, which may not always be true. Fourth, information about the frequency of UAI and PAI was not collected in this study. The design allows for investigation of event-specific factors among inconsistent condom users. However, the design also treated inconsistent condom users with NRP as a homogeneous group while inconsistent condom users with NRP may in fact vary in frequency of intercourse and frequency of UAI. Fifth, some limitations exist with regard to measurements. In this study, we asked whether the participants felt nervous in general prior to the last episode of UAI or PAI of concern. We did not ask the participant to specify the reasons for their nervous feeling and whether this feeling was related to condom use, but it would be difficult to discern the cause of such feelings. The variable of sexual dysfunction referred to perceived sexual performance in the last month; the situation may vary over time but it might not be strictly regarded as an event-specific situational factor. Another limitation was related to variable selection. Our variable selection was based on a literature search of traditional cross-sectional studies as the few case-crossover studies on HIV-related behaviors used only a narrow range of independent variables [38, 39]. In addition, some inconsistent condom users may not admit their condom use status or the fact that they had had sex with NRP due to social desirability. Selection bias may also exist due to the absence of a sampling frame, although previous studies on HIV-related behaviors among MSM have also used similar mixed-method recruitment strategies [18–20, 40, 41].

In sum, we found that availability and possession of condoms, condom negotiation, and planning to use condoms were event-specific protective factors against episodes of UAI with NRP among MSM who were inconsistent condom users. We also discovered some new situational risk factors such as anal sex taking place during the weekday and tiredness prior to anal sex and have identified other situational and environmental protective or risk factors such as display of reminders promoting condom use, home settings, travel, and nervous feelings. We have further found that factors such as the physical environments, particular sexual behaviors, and assessment of the partner's risk were nonsignificant. These findings provide important insights for designing new HIV prevention targeting inconsistent condom users by putting more emphasis on event-specific factors. Such factors may partially explain why condoms are sometimes but not always used by MSM.

## Figures and Tables

**Table 1:** Background characteristics of participants.

	All	Recruited from gay venues	Recruited from internet	*P* value^#^
	% (*n* = 215)	% (*n* = 151)	% (*n* = 64)	
*Sociodemographic characteristics *				
Age (years)				
≤30	53.0	49.7	60.9	
31–40	34.4	34.4	34.4	
41–50	9.3	11.3	4.7	
≥51	3.2	4.6	0.0	0.105
Education level				
Junior high or lower	7.4	7.9	6.3	
Senior high	29.8	31.1	26.6	
College and above	62.8	60.9	67.2	0.681
Current marital status				
Single	87.4	86.0	92.2	
Cohabiting with a man	7.9	8.0	7.8	
Cohabiting with or married to a woman	3.8	5.3	0.0	
Divorced/widowed/others	1.0	0.7	0.0	0.257
Sexual orientation				
Homosexual	87.4	87.4	87.5	
Bisexual	10.7	9.9	12.5	
Heterosexual/uncertain	1.9	2.6	0.0	0.373
Mode of recruitment				
Bar or disco	30.2			
Sauna	35.8			
Internet	29.8			
Beach	4.2			

^#^By chi-square test.

**Table 2:** Frequency distribution of the nonregular sex partners' (NRP) attributes.

	Episode involving condom use (i.e., PAI)	Episode involving no condom use (i.e., UAI)
	(*n* = 215)	(*n* = 215)
	Col %	Col %
*Sociodemographic characteristics *		
Age (years)		
≤25	23.7	31.2
25–35	45.1	41.9
36–45	9.8	11.2
>46	4.7	5.1
Do not know	16.7	10.6
Where is he from?		
Hong Kong	82.8	82.8
Mainland	4.7	7.4
Other countries	11.2	8.4
Do not know	1.4	1.4
*Sexual experiences *		
How long did you know him before the intercourse?		
On the same day	55.8	59.5
<0.5 months	19.5	20.5
0.5–2 months	10.2	8.8
2–6 months	5.6	4.7
>6 months	8.8	6.5
Times of anal sex with the partner before		
0 times	66.0	71.2
1-2 times	20.5	21.4
≥3 times	13.5	7.4
Did you and your sex partner have equal sex experience?		
About the same/do not know	47.4	58.2
More experience	35.8	26.0
Less experience	16.7	15.8
*Style of the partner *		
Physical appearance of the sex partner		
Neutral	38.1	44.7
Masculine	47.9	47.0
Feminine	14.0	8.3
Personality of the sex partner		
Passive	22.8	14.9
Assertive	50.2	48.8
Neutral	26.0	34.9
Do not know	0.9	1.4
*Liking and relationship *		
Attractiveness of the sex partner		
Very attractive/attractive	87.0	89.8
Not attractive/not very attractive	12.5	9.7
Do not know	0.5	0.5
How much did you like the sex partner (0–10)?		
1–3	3.3	5.6
4–6	28.8	20.9
≥7	64.7	72.6
Do not know	3.3	0.9
How much did the sex partner like you (0–10)?		
1–3	1.9	4.2
4–6	17.2	24.6
≥7	65.6	52.6
Do not know	15.3	18.6
Degree participant liked the sex partner more (difference in 2 likeliness scores)^#^		
<−3	1.5	1.9
−1 to −3	20.9	17.6
0	40.0	38.1
1 to 3	20.9	20.5
>3	1.4	3.3
Do not know	15.3	18.6

^#^It is the difference between the two likeliness scores: the degree of participant like the sex partner minus the degree of the sex partner like the participant.

**Table 3:** Frequency distributions of the situational variables.

	Episode involving condom use (i.e., PAI)	Episode involving no condom use (i.e., UAI)
	(*n* = 215)	(*n* = 215)
	Col %	Col %
*Spirit prior to the episode of anal sex *		
Mood prior to the episode		
Very good/good	50.2	47.4
Normal	44.2	45.1
Bad/very bad	2.3	3.3
Do not know/cannot remember	3.3	4.2
Tiredness prior to the episode		
Very energetic/energetic	46.0	42.4
Normal	45.2	42.8
A bit tired/very tired	6.0	10.7
Do not know/cannot remember	2.8	4.2
Were you (the participant) nervous prior to the sex episode?		
Not at all	47.0	39.5
Not really	39.1	36.3
A bit/very much	11.2	20.9
Do not know	2.8	3.3
*Risk assessments *		
What do you think about the current risk of HIV infection of your sex partner?		
Not infected	43.7	45.6
Already infected/very high risk/high risk	5.1	4.7
Very low risk/low risk	51.2	49.7
What do you think about the current risk of STI of your sex partner?		
Not infected	43.3	44.7
Already infected/very high risk/high risk	3.7	5.6
Very low risk/low risk	53.0	49.7
Did his penis and anus look clean?		
Yes	73.5	70.7
How many male sex partners do you think your sex partner had in the last month?		
None	20.9	17.2
1–5	29.8	28.8
6–10	3.7	6.0
>10	1.9	1.9
Do not know	43.7	46.0
How likely did you think he would use condoms with other male sex partners?		
Very likely/moderate/no other partner	58.6	50.2
Not likely/never	41.4	49.8
One week before intercourse, did you have UAI with any other male partner?		
Yes	20.9	14.4
Did you and your sex partner have a third common sex partner?		
Yes	7.0	8.4
*Communication & planning about condom use *		
Did you discuss condom use with the partner before UAI/PAI?		
Yes	37.2	21.4
Did sex partner suggest using a condom?		
Yes	34.0	16.3
Did sex partner suggest NOT using a condom?		
Yes	13.0	24.2
Did you suggest using a condom?		
Yes	43.7	20.0
Did you suggest NOT using a condom?		
Yes	6.5	15.3
Did you plan to use a condom during that sexual encounter?		
Yes	81.9	54.9
Did you perceive that the sex partner would like to use a condom?		
Yes	50.7	15.8
*Particular sexual behavior *		
Degree of sexual compulsivity of participant (0–10)		
1–5	9.4	6.6
6–10	90.6	93.4
Degree of sexual compulsivity of sex partner (0–10)		
1–5	17.2	12.1
6–10	82.8	87.9
Participant's sexual position		
Insertive	46.0	46.5
Receptive	37.7	33.0
Both	14.9	20.5
Do not know	1.4	0
One month before the episode, did you have any problem with sexual dysfunction?		
Yes	6.0	7.4
During the intercourse, how many sex partners were there?		
1	96.7	94.4
More than 1	3.3	5.6
Any erotic activities involved?		
Yes (at least one)∗	26.0	22.3
Whether money transaction was involved in the episode?		
No	97.7	96.7
Yes	2.3	1.9
Cannot remember	0	1.4
*Substance use *		
Whether you (the participant) or sex partner drank alcohol before the anal sex episode? (if either man drank answer is classified as “yes”)		
Yes	17.2	18.6
Whether you or sex partner used Viagra or psychoactive substances before the anal sex episode?		
Yes	2.3	4.2

*Erotic activities include watching sex movie before UAI, reading pornographic magazines, the use of sex toys, SM behaviour, and wearing special outfits.

**Table 4:** Frequency distributions of environmental/settings variables.

	Episode involving condom use (i.e., PAI)	Episode involving no condom use (i.e., UAI)
	(*n* = 215)	(*n* = 215)
	Col %	Col %
*Timing and location *		
Location of the episode		
Hong Kong	89.3	90.2
Overseas	10.7	9.8
Happened during a weekend?		
Weekend	46.0	32.1
Weekday	54.0	67.9
Hour of the day when it occurred?		
Morning/afternoon	32.1	30.2
Evening	67.9	69.8
Did the episode occur at home setting?		
Yes (participant or sex partner's home)	34.4	29.7
No (e.g., hotel, sauna, etc.)	65.5	70.2
*Availability of condoms *		
Were condoms already available at the venue?		
Yes	81.9	70.7
Were there free condoms available at the venue?		
Yes	60.9	56.7
Were there any notices (e.g., posters) promoting condom use at the venue then?		
Yes	49.3	41.4
Were you carrying a condom with him?		
Yes	37.2	29.3
Was the sex partner carrying a condom with him?		
Yes	23.3	11.6
*Physical environment *		
Was it a dark environment?		
Yes	70.2	68.4
Was there a place for shower?		
Yes	97.7	96.7
Was the hygiene relatively poor?		
Yes	7.0	7.9

**Table 5:** Factors associated with UAI involving male NRP.

	Episode involving condom use	Episode involving no condom use	Matched OR
	(*n* = 215)	(*n* = 215)	
	Row %	Row %	
*Sociodemographic characteristics *			
Age (years)			
≤25	43.2	56.8	1
26–35	51.9	48.1	0.52 (0.28–0.97)∗
36–45	46.7	53.3	0.66 (0.26–1.72)
>46	47.6	52.4	0.93 (0.13–6.50)
Do not know	61.0	39.0	0.29 (0.12–0.70)∗∗
*Sexual experiences *			
Times of anal sex with the sex partner before			
0 times	48.1	51.9	1
1-2 times	48.9	51.1	1.02 (0.49–2.12)
≥3 times	64.4	35.6	0.28 (0.10–0.75)∗
Did your sex partner have equal sex experience as you?			
About the same/do not know	44.9	55.1	1
More experience/less experience	55.7	44.3	0.47 (0.27–0.79)∗∗
*Style of the partner *			
Physical appearance of sex partner			
Neutral	46.1	53.9	1
Masculine	50.5	49.5	0.67 (0.38–1.20)
Feminine	62.5	37.5	0.16 (0.04–0.57)∗∗
Personality of sex partner			
Passive	60.5	39.5	1
Assertive	50.7	49.3	2.18 (1.07–4.45)∗
Neutral	42.7	57.3	4.30 (1.81–10.17)∗∗∗
Do not know	40.0	60.0	4.48 (0.35–57.46)
*Liking *&* relationship *			
How much did the sex partner like you?			
1–3	30.8	69.2	1
4–6	41.1	58.9	0.60 (0.14–2.69)
≥7	55.5	44.5	0.19 (0.04–0.81)∗
Do not know	45.2	54.8	0.76 (0.17–3.47)
*Spirit prior to the episode of anal sex *			
Tiredness before the episode			
Very energetic/energetic/normal	51.7	48.3	1
A bit tired/very tired	36.1	63.9	2.73 (1.07–7.00)∗
Do not know/cannot remember	40.0	60.0	1.61 (0.57–4.56)
Were you (the participant) nervous prior to the sex episode?			
Not at all	54.3	45.7	1
Not really	51.9	48.1	1.33 (0.75–2.33)
A bit/very	34.8	65.2	3.32 (1.57–7.06)∗∗
Do not know	46.2	53.8	1.61 (0.47–5.57)
*Risk assessments *			
One week before intercourse, did you have UAI with any other male partner?			
No	48.0	52.0	1
Yes	59.2	40.8	0.39 (0.18–0.85)∗
How likely did you think he would be to use condoms with other male sex partners?			
Very likely/moderate/no other partners	53.8	46.2	1
Not likely/never	45.4	54.6	2.29 (1.22–4.28)∗
*Communication & planning about condom use *			
Did you discuss condom use with partner before UAI?			
No	44.4	55.6	1
Yes	63.5	36.5	0.29 (0.16–0.53)∗∗∗
Did you perceive that the sex partner would like to use a condom?			
No	36.9	63.1	1
Yes	76.2	23.8	0.11 (0.05–0.21)∗∗∗
Did your sex partner suggest using a condom?			
No	44.1	55.9	1
Yes	67.6	32.4	0.30 (0.17–0.52)∗∗∗
Did your sex partner suggest NOT using a condom?			
No	49.0	51.0	1
Yes	35.0	65.0	4.00 (1.84–8.68)∗∗∗
Did you plan to use a condom?			
No	28.7	71.3	1
Yes	59.9	40.1	0.16 (0.08–0.30)∗∗∗
Did you suggest using a condom?			
No	41.3	58.7	1
Yes	68.6	31.4	0.19 (0.10–0.35)∗∗∗
Did you suggest NOT using a condom?			
No	52.5	47.5	1
Yes	29.8	70.2	2.90 (1.41–5.95)∗∗
*Timing *&* location *			
Happen during weekend?			
Weekend	58.9	41.1	1
Weekday	44.3	55.7	2.50 (1.49–4.20)∗∗∗
*Availability of condoms *			
Were condoms already available at the venue?			
No	38.2	61.8	1
Yes	53.7	46.3	0.40 (0.22–0.71)∗∗
Were there any notices (e.g., posters) promoting condom use at the venue then?^ #^			
No	46.4	53.6	1
Yes	54.4	45.6	0.40 (0.21–0.76)∗
Were you carrying a condom?			
No	47.0	53.0	1
Yes	55.9	44.1	0.45 (0.24–0.85)∗
Was the sex partner carrying a condom with him?			
No	46.5	53.5	1
Yes	66.7	33.3	0.27 (0.13–0.56)∗∗∗

*P < 0.05; **P < 0.01; ***P < 0.001.

^#^Adjusted for location of sex episode.
